# Supplementary material for: Enhancing Visual Exploration through Augmented Gaze: High Acceptance of Immersive Virtual Biking by Oldest Olds
Source: Int J Environ Res Public Health. 2023 Jan 17;20(3):1671. doi: 10.3390/ijerph20031671 (PMC9914324; doi:10.3390/ijerph20031671)
Supplement: Supplementary file 1 [file ijerph-20-01671-s001.zip › ijerph-2119054-supplementary.pdf]

Supplementary Material

**Combined UX**	*Slope*	*Lower bound*	*Upper bound*	*T-stat*	*Degrees of freedom*	*P-value*
AGE	0.026	-0.016	0.068	1.644	51	0.106
GENDER	-0.325	-1.008	0.358	-1.272	51	0.209
MMSE	-0.014	-0.073	0.045	-0.634	51	0.529
TINETTI	0.024	-0.029	0.077	1.213	51	0.231
STAY	0.010	-0.008	0.028	1.497	51	0.141
GAIN	-0.060	-0.313	0.194	-0.631	51	0.531
SESSION DUR	0.001	-0.002	0.003	0.866	51	0.391

Table S1. Results of the mixed-models analysis on the combined user experience (UX) index. Lower bound and Upper bound are the 99% confidence intervals of the Slope. Statistical significance at the 0.01 level is highlighted in bold. A p-value of 0.000 means significance below 0.0005.

**SSQ**	*Slope*	*Lower bound*	*Upper bound*	*T-stat*	*Degrees of freedom*	*P-value*
AGE	0.151	-0.416	0.718	0.711	51	0.480
GENDER	0.920	-8.300	10.141	0.267	51	0.791
MMSE	-0.203	-0.997	0.592	-0.682	51	0.498
TINETTI	0.192	-0.526	0.909	0.714	51	0.478
STAY	0.009	-0.236	0.254	0.101	51	0.920
GAIN	-0.130	-3.811	3.551	-0.095	51	0.925
SESSION DUR	-0.003	-0.039	0.033	-0.215	51	0.830

Table S2. Same as Table S1 but for SSQ score.

**Session duration**	*Slope*	*Lower bound*	*Upper bound*	*T-stat*	*Degrees of freedom*	*P-value*
AGE	0.039	-0.114	0.191	0.680	52	0.500
GENDER	-0.706	-3.235	1.822	-0.747	52	0.459
MMSE	-0.003	-0.221	0.215	-0.036	52	0.972
TINETTI	0.005	-0.191	0.202	0.072	52	0.943
STAY	-0.004	-0.070	0.063	-0.153	52	0.879
GAIN	0.168	-0.662	0.998	0.541	52	0.591

Table S3. Same as Table S1 but for session duration.

**Pedalling duration**	*Slope*	*Lower bound*	*Upper bound*	*T-stat*	*Degrees of freedom*	*P-value*
AGE	-2.721	-10.243	4.801	-0.967	52	0.338
GENDER	15.057	-109.684	139.798	0.323	52	0.748
MMSE	-5.801	-16.542	4.940	-1.444	52	0.155
TINETTI	0.204	-9.473	9.880	0.056	52	0.955
STAY	0.562	-2.673	3.797	0.465	52	0.644
GAIN	-19.992	-52.821	12.837	-1.628	52	0.110

Table S4. Same as Table S1 but for pedalling duration.

**Head rotation**	*Slope*	*Lower bound*	*Upper bound*	*T-stat*	*Degrees of freedom*	*P-value*
AGE	-0.020	-0.046	0.006	-2.018	51	0.049
GENDER	0.425	0.022	0.828	2.822	51	**0.007**
MMSE	-0.016	-0.051	0.019	-1.229	51	0.225
TINETTI	0.005	-0.026	0.037	0.434	51	0.666
STAY	-0.004	-0.015	0.008	-0.870	51	0.388
GAIN	-0.219	-0.345	-0.093	-4.652	51	**0.000**
SESSION DUR	0.001	-0.001	0.002	1.304	51	0.198

Table S5. Same as Table S1 but for head rotation.

**Visual shift**	*Slope*	*Lower bound*	*Upper bound*	*T-stat*	*Degrees of freedom*	*P-value*
AGE	-0.018	-0.045	0.009	-1.822	51	0.074
GENDER	0.420	0.002	0.837	2.691	51	0.010
MMSE	-0.015	-0.051	0.021	-1.103	51	0.275
TINETTI	0.003	-0.029	0.036	0.274	51	0.785
STAY	-0.004	-0.015	0.008	-0.828	51	0.412
GAIN	0.334	0.202	0.467	6.763	51	**0.000**
SESSION DUR	0.001	-0.001	0.002	1.439	51	0.156

Table S6. Same as Table S1 but for visual rotation.
